# Development of Body-Tissue Temperature-Control Transducer

**DOI:** 10.3390/s19010014

**Published:** 2018-12-20

**Authors:** Audrone Dumciene, Saule Sipaviciene

**Affiliations:** 1 Department of Health, Physical and Social Education, Lithuanian Sports University, Sporto St. 6, LT-44221 Kaunas, Lithuania; 2 Department of Applied Biology and Rehabilitation, Lithuanian Sports University, Sporto St. 6, LT-44221 Kaunas, Lithuania; saule.sipaviciene@lsu.lt

**Keywords:** body temperature, sensors, transducer

## Abstract

The aim of this study was to develop a transducer for non-invasive temperature measurement in deeper tissue layers during tissue cooling. Simulation of the temperature field distribution in human tissues and the transducer were done, and the influence of transducer structure and material properties were studied. Using simulation results, the experimental transducer was designed for temperature measurement in deeper tissue layers during cooling. The temperature measurements with the needle thermometer and the transducer were well correlated at both before tissue cooling *r* = 0.723 and after cooling *r* = 0.945, and the temperature difference was no more than ±0.2 °C.

## 1. Introduction

Several concepts are used in human-body temperature measurements—core body temperature, body temperature, muscle temperature, body skin temperature, etc.

The functioning of body-temperature measuring devices is based on various physics principles (nonelectric, thermoelectric, resistance, impedance, semiconductors, fiberoptic, ultrasonic, etc.). There is no rigorous classification of body-temperature measurement methods and gear due to the invasiveness. Gear can be divided into invasive, if an invasion in the human body is necessary to over-extend the skin; less-invasive, if natural body openings are used for temperature measurement; and non-invasive, if body temperature is measured on intact skin surface [1,2].

Core-body temperature can be most accurately measured with a gear known as the Swan–Ganz catheter, which is invasively introduced into the pulmonary artery, or by an esophageal catheter inserted into the esophagus [3]. The core-body temperature of healthy people is in the range of 37 °C ± 0.6 °C, which can be changed with the circadian rhythm. This temperature is assumed to be relatively standard, and it is called the “gold standard.” Invasive temperature measurements in deeper layers of tissue are performed using needle thermometers (thermocouple) [4,5,6].

For precise less-invasive body-temperature measurements, tympanic ear, oral, and rectal thermometers are more commonly used [7,8,9]. Some studies have shown that the normal body temperature, measured by less-invasive body-temperature methods, can be in the range of 36.2 °C to 37.5 °C [10].

The advantages and disadvantages of invasive and less-invasive methods have been discussed [1], stating that invasive (pulmonary artery, esophagus, and nasopharynx) methods are only clinically relevant and associated with certain risks, while less-invasive (urinary bladder and rectum) methods are less accurate [1].

In the absence of a commonly agreed classification of body-temperature measurement methods, some researchers assign methods and gears to non-invasive if the natural body openings are used for temperature measurement [11,12,13,14,15,16].

Non-invasive measurements of human body temperature utilize various digital medical thermometers, in which thermistors are commonly used as temperature sensors. Known as “zero-heat-flow” (ZHF) temperature gauges, they come with the following options—a heat source to create a heat-flow equilibrium between the human body and an artificial heat source [3,17,18], two sensors that do not require a heat source [19,20,21], and four temperature transducers [22], which have been used in various studies [23]. Magnetic Resonance Imaging (MRI) [24] and the multi-frequency impedance method [25] are used to monitor the distribution of temperature fields in tissue.

The most common tools used for measuring skin temperature are contactless infrared and conductive devices [26,27]. There are commercial non-invasive body temperature ZHF sensors, with a declared accuracy of 25 °C to 43 °C ± 0.2 °C [28].

A comprehensive comparative study of invasive (a urinary bladder temperature probe), semi-invasive (in the esophagus during vascular surgery and in the nasopharynx and the pulmonary artery during cardiac surgery), and non-invasive measurements of temperature using zero-heat-flux sensor type SpotOn skin probe on the forehead and skin temperature probe on the forehead was conducted [1]. The results of this study showed a good agreement (95% limit of agreement) between zero-heat-flux sensor body measurements and invasive measurements. But there was a bad coincidence when the body temperature was below 32 °C. The forehead skin temperature was “2 to 30 °C lower than the deep body temperature” [1] (p. 976). Thus, according to Reference [1], the SpotOn skin probe is suitable for body temperature and core temperature measurements alongside invasive methods until the body temperature is higher than 32 °C.

Body-temperature measurements by non-invasive methods, the author’s so-called skin-temperature measurement methods, are extensively analyzed in a review article [29]. It states that, in their opinion, there is no universally acceptable method of measurement to measure the temperature of the skin. The measurement results can be influenced by many factors such as sensor properties, clamping, and attaching to a particular body, environmental conditions, and other factors. It is likely that the thermal balance between the body’s area covered with a sensor and its environment results in heat-transfer conditions different from that in the adjacent uncovered skin zones, and the measured temperature should usually differ from the temperature of the adjacent areas of the skin.

For measuring temperature in deeper tissue layers, needle thermometers are usually used, which are inserted 2–4 cm in depth [6,30,31,32,33,34,35,36,37]. They must be sterilized before use and disinfected after use. This measurement procedure is not pleasant to the subject.

The above-discussed methods and gears were used to measure body, body core, and skin temperature, but we did not find any research that proves that these methods and gears could be used to determine the temperature at a certain tissue level. This is required for the research on the effects of cooling on muscle properties [31,32], or of cooling for treatment [4,6,32,33,34,35,36,37].

The temperature in deeper tissue layers was measured by microwave radiometry in which the tissues were heated by high-intensity-focused ultrasound, and using temperature imaging algorithm, the temperature distribution was calculated in real time [38], and microwave radiometry was used for the brain temperature measurement [39]. The mathematical model that describes the relationships of the core temperature with the individual physical activity, heart rate, skin temperature, ambient temperature, and relative humidity was proposed in a previous study [40]. An algorithm was developed for calculating the core temperature from the measurements data mentioned above. In Reference [41], a model to calculate core body temperature from heart rate observation data was proposed, but it needed continuous heart rate monitoring. Mathematical models [42] enabling assessment of core-body temperature using individual characteristics, physical activity, clothing biophysics, and environmental conditions were created. A model was proposed in study [43] for core-body temperature prediction using the skin temperature over the carotid artery measurement data. New design for non-invasive core temperature measurement using ZHF method was proposed and used in Reference [44]. Intramuscular temperature can be predicted at a depth of 2 cm using the equation proposed in Reference [33], which includes variables like time, skin temperature, skinfold, room temperature, and body core temperature. Temperature was measured at the given tissue depth using diffuse optical spectroscopic imaging method and a relevant equipment [45]. A method of temperature estimation that was based on the thermal dependence of the acoustic speed in a heated medium was used in Reference [46]. Novel photoacoustics sensor was proposed and used in study [47] for non-invasive monitoring of blood temperature.

The methods implemented in References [38,45,46] hold a possibility for temperature measurement in tissue deeper layers and in local areas unlike the methods used in References [40,41,42,43,44,47] for measuring core body temperature, but the equipment based on the diffuse optical spectroscopic imaging method or the thermal dependence of the acoustic speed in a heated medium is complicated to use in additional cases, for example, when treating with an ice pack after injury or trauma.

It was shown in Reference [31] that skin surface temperature during cooling versus time change almost linearly. It was found in Reference [6] that when the surface (skin) temperature varied linearly in time during cooling, temperature of muscles at 2 cm depth also varied linearly with time. So, if it was possible to measure the temperature of the tissue surface area, thermo-isolated from the thermal effects of the environment, then the tissue temperature at a depth of 3 cm can be calculated using the following equation [48]:

*T* = 0.5026 × *T*_ss_ + 18.399 (1)

where *T*_ss_—temperature on the skin surface measured by the transducer.

Therefore, the purpose of this study was to find out the possibility and develop an instrument for measuring temperature in deeper layers of tissue, without the use of needle thermometers, but by measuring temperature on intact skin surface during cooling.

## 2. Materials and Methods

**Figure 1:**
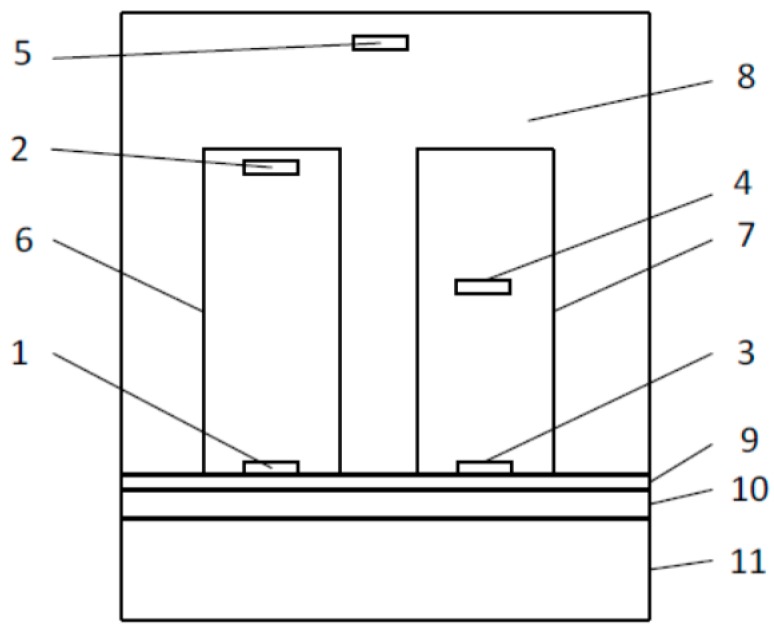
Transducer structure: 1, 2, 3, 4, and 5—temperature sensors (Thermistor 5 can be used to inspect the thermo-isolation cover); 6—thermal channel; 7—thermal channel; 8—thermo-insulation cover; 9—skin; 10—fat; and 11—muscle.

Here, we discuss a new multisensory transducer design, of which the structure is shown in Figure 1. This is because the thermal channel and isolation cover dimension and the influence of the channel material have not been clarified.

**Figure 2:**
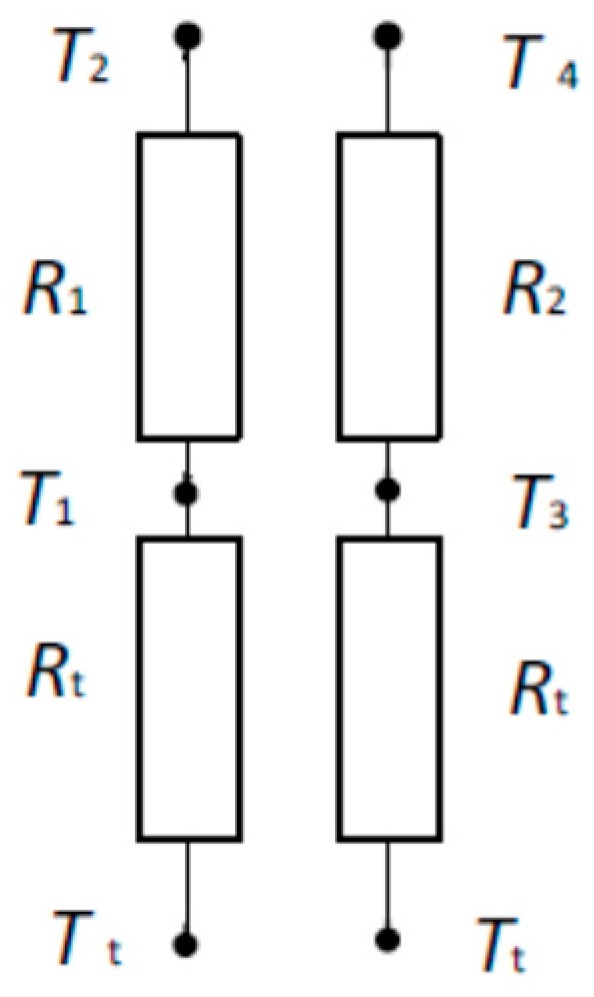
Equivalent resistance circuit of two heat-flow channels. *T*_t_—deep tissue layer temperature; *T*_1_—sensor 1 temperature; *T*_2_—sensor 2 temperature; *T*_3_—sensor 3 temperature; *T*_4_—sensor 4 temperature; *R*_t_—tissue thermal resistance; *R*_1_—thermal resistance of one thermal channel; and *R*_2_—thermal resistance of another thermal channel.

The thermal equivalent circuit of the transducer shown in Figure 1 without considering the influence of the thermistors can be formed, as shown in Figure 2.

The temperature on the boundary between the skin surface and the transducer pad can be calculated using the following modified equation [48]:

(2) $$T_{t}=\frac{T_{1}\cdot \lambda (T_{3}-T_{4})-T_{3}(T_{1}-T_{2})}{\lambda (T_{3}-T_{4})-(T_{1}-T_{2})}$$

where *T*_1_, *T*_2_, *T*_3_, and *T*_4_—temperatures at points shown in Figure 2; and *λ*—ratio of thermal channel thermal resistance *R_1_/R_2_.*

This ratio *λ* must be higher than four [48]. So, the ratio of thermal resistors *R*_1_ and *R*_2_ of the thermal channels in this study was equal to five.

To determine the optimum parameters of the transducer, a simulation of the distribution of temperature fields in the human tissue and the transducer structure was performed.

The human body, using nutrients, generates bioheat. Due to the flow of blood and the heat-transfer process, heat is transferred to the entire body until it is balanced with the environment. The description of heat propagation in living organisms is most often done with the Pennes’ [49] equation or its modifications, which assess specific conditions [50,51,52]. Using the Pennes’ equation and the boundary conditions established for the variants under consideration, one can simulate the heat-propagation process in the human body and calculate the distribution of temperature fields.

Thus, the bioheat-transfer process in the human body can be described by the following equation [49]:

(3) $$\delta_{ts}\rho C\frac{\delta T}{\delta t}+\nabla \cdot (-k\nabla T)=\rho_{b}C_{b}\omega_{b}(T_{b}-T)+Q_{m}+Q_{ex}$$

where *δ_ts_*—a time-scaling coefficient (dimensionless); *ρ*—tissue density (kg/m^3^); *C*—specific heat of tissue (J/(kg·K)); *k*—tissue’s thermal conductivity tensor (W/(m·K)); *ρ_b_*—blood density (kg/m^3^); *C_b_*—specific heat temperature (K); *Q_m_*—heat source from metabolism (W/m^3^); *Q_ex_*—spatial heat sourse in body of blood (W/m^3^); *ω_b_*—blood-perfusion rate (m^3^/(m^3^·s)); *T*_b_—arterial blood temperature (K); *T*—dependence variable temperature (K).

We investigated a case where the person is in a state of rest and at a relatively stable ambient temperature. In this case, we can write:

(4) $$\nabla \cdot (-k\nabla T)=\rho_{b}C_{b}\omega_{b}(T_{b}-T)+Q_{m}+Q_{ex}.$$

If the person is in a state of rest, we can accept that *Q_ex_* = 0, and transform Equation (4) as

(5) $$\nabla \cdot (-k\nabla T)=\rho_{b}C_{b}\omega_{b}(T_{b}-T)+Q_{m}.$$

Therefore, Equation (4) can be used to simulate temperature fields in the tissue. We are interested in the distribution of temperature fields in the tissue and in the transducer body.

Since the volume of the transducer is small compared with the parts of the human body in which the temperature will be measured, the accuracy is reduced using Equation (5) for the propagation of heat in the transducer body.

Boundary conditions: $-\vec{n}\cdot (-k\nabla T)=q_{0}+h(T_{ext}-T)$—heat flux (external boundary of the tissue and the transducer model); $\vec{n}\cdot (k_{1}\nabla T_{1}-k_{2}\nabla T_{2})=0$—continuity on the entire interior boundary between tissue layers and the transducer body model; $-\vec{n}\cdot (-k\nabla T)=0$—using the symmetry of the object under study, the symmetry condition is given, where *T_ext_*—external (bulk) temperature.

The human tissue model properties [53] used in the simulation are given in Table 1.

The COMSOL Multiphysics 3.5. software package was used to simulate temperature fields in the tissue and transducer body at an environment temperature of 20 °C.

Precision Cantherm thermistor (Cantherm, Monteal, Canada), type MF51E103F3380, with a time constant of ≤3.2 s was used as temperature sensor that is intended for medical equipment. For the thermistor, a calibration procedure was conducted, and the Steinhart–Hart equation was used for the resistance temperature *R*(*T*) characteristic approximation. The equation can be written as follows:

(6) $$1/T=B_{0}+B_{1}\ln R_{T}+B_{3}{\left(\ln R_{T}\right)}^{3}$$

where *T*—temperature in K; *B*_0_, *B*_1_, and *B*_3_—equation coefficients; and *R_T_*—thermistor resistance at the temperature *T.*

Coefficients *B*_0_, *B*_1_, and *B*_3_ were estimated from thermistor calibration results, since the *R(T)* characteristics from the manufacturer are not for individual thermistors, but averaged thermistor type MF51. The accuracy of calibrated thermistors after applying the linearization of the Steinhart—Hart equation was in the range of ±0.02 °C.

**Figure 3:**
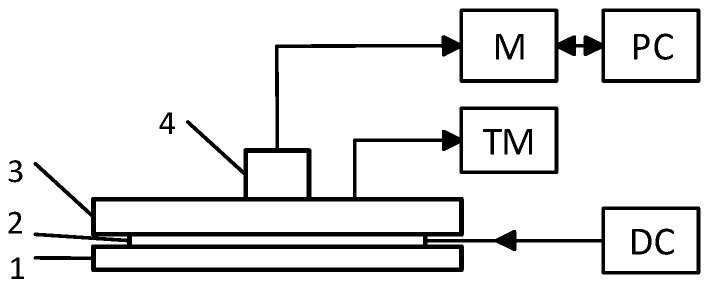
Structure diagram of the experiment. 1—cooler; 2—Peltier element; 3—polyethylene plate (phantom); 4—transducer; M—temperature meter; TM—temperature meter with thermo-probe; PC—laptop; and DC—programmable DC source.

The structure of the physical model used for transducer examination is shown in Figure 3.

Temperature was measured at six points using the FLUKE thermometer type Black Stack 1560 and Thermistor readout module type 2564 (readout four decimal digits after point). Reference temperature at the top of the polyethylene plate (phantom) was maintained within the range of 30 ± 0.1 °C.

The temperature of transducer sensors 1, 2, 3, 4, and 5 was measured using, as noted above, a FLUKE thermometer.

**Figure 4:**
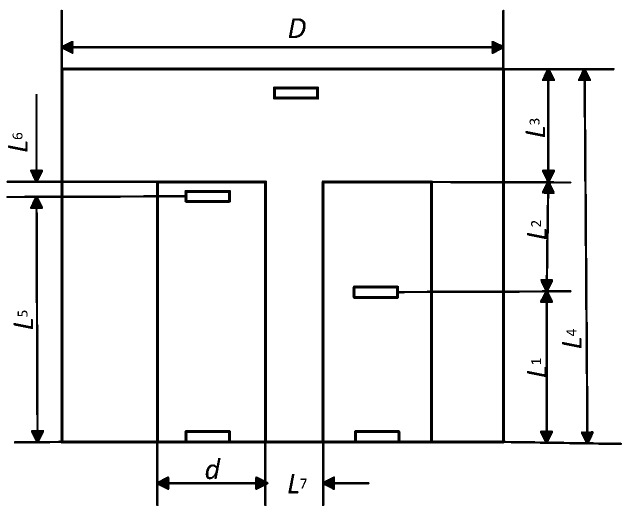
Main dimension of transducer elements.

The dimension of the thermal channel and the thermos-isolation cover is shown in Figure 4.

Dimensions L_1_, L_2_, L_3_, L_4_, L_5_, L_6_ L_7_, D, and d shown in Figure 4 are used as variables by simulating the distribution of temperature fields in a transducer.

A hard-plastic cover and polyurethane foam for thermo-isolation and epoxy with cooper powder were used for the transducer design.

Ethical approval for an experiment with human subjects was granted by the Kaunas regional biomedical-research ethics committee, and measurements were taken in accordance with the Helsinki Declaration.

The experiment with the human subject included one volunteer. Before temperature measurements were taken, the test subject was lying supine on a laboratory bed for 15 min for relaxation in a room with a temperature of 20 ± 1 °C. The deep muscle temperature was measured using needle thermometer (Ellab A/S, type DM 852, Hillerød, Denmark). The skin of left thigh was prepared before each measurement by shaving and disinfecting before and after needle insertion with a pad saturated with isopropyl alcohol (Shanghai Channelmed Import & Export Co., Ltd., Shanghai, China). Needle thermometer was inserted perpendicular to the skin covering of the muscle quadriceps femoris at a depth of ~3 cm including one-half of the skinfold in the middle-third on the side of the femur. The skinfold was measured by the Holtain caliper (Siber Hegner GMP, Zurich, Switzerland). After measuring, the needle thermometer was extracted, and the skin was disinfected and sealed with a waterproof anti-bacterial patch on the spot.

Transducer was hermetically attached using adhesive tape 1 cm away from the needle thermometer and left during immersion in cold bath. The needle thermometer was sterilized after each use. The measurements were conducted before and repeated immediately after leg cooling. Test subject’s leg was twice immersed for 15 min (with a 10 min break) in cold water bath (15 ± 1 °C) for cooling [54]. The measurement was repeated five times with two-week breaks. The patient was advised on the thermometer needle insertion site supervision and to inform researchers about any signs of infection.

## 3. Results

**Figure 5:**
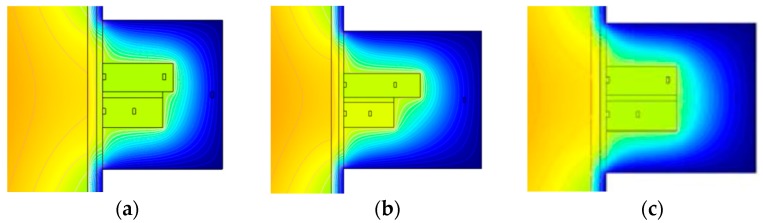
Distribution of temperature fields in the tissue and the transducer. (**a**) Transducer structure version when (L_1_ + L_2_) < (L_5_ + L_6_); (**b**) Transducer structure version when (L_1_ + L_2_) ≪ (L_5_ + L_6_); (**c**) Transducer structure version when (L_1_ + L_2_) = (L_5_ + L_6_) and L_3_ is higher than in versions (**a**,**b**). Variables L_7_ and d in all three versions are constant.

The distribution of the temperature fields in the tissue–transducer models (Figure 1), at different variables L_1_, L_2_, L_3_, L_4_, L_5_, and L_6_, when the environment temperature was +20 °C, is given in Figure 5 (a, b, and c for different variable values).

**Figure 6:**
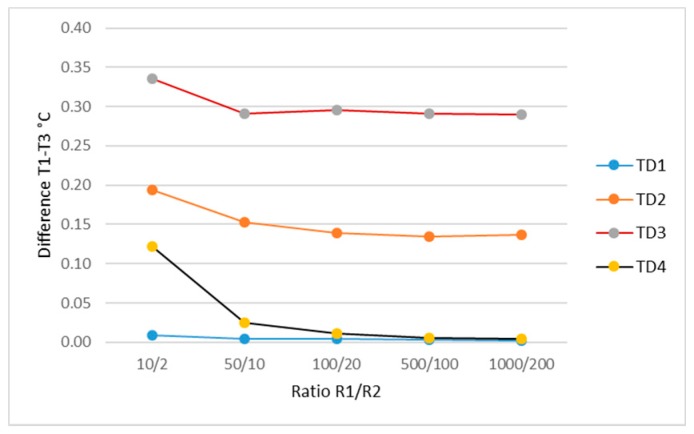
Influence of thermal channels materials’ thermal conductivity on the material temperature at the boundary between the tissue surface and the transducer pad.

The simulation results of the influence of thermal channels materials’ thermal conductivity in the temperature on the boundary between the tissue surface and the transducer pad are shown in Figure 6.

In the evaluation of simulation results, from Figure 6, it can be concluded that for the purpose of obtaining a lower transductor temperature time constant, the materials for a thermal channel are required to have a thermal conductivity of 500/100–1000/200 (where the thermal-conductivity coefficient of the short channel material is in a fraction denominator, and of the longer channel in a counter).

**Figure 7:**
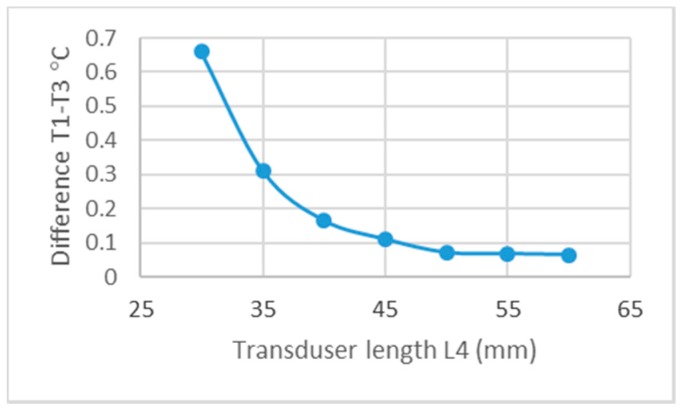
Influence of length *L*_4_ on the *T*_1_–*T*_2_ difference.

The optimization results of thermal-insulation cover thickness L_3_ (Figure 4) as a function of different temperatures *T*_1_ and *T*_3_ for illustration are shown in Figure 7.

**Figure 8:**
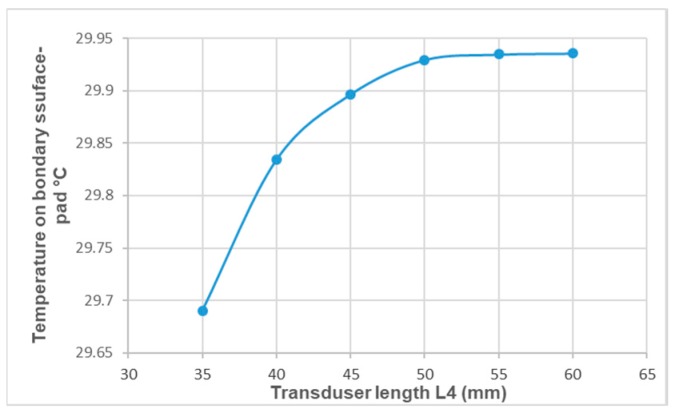
Influence of length *L*_4_ on the temperature on the boundary between the tissue surface and the transducer pad.

The simulation results of the influence of the thermal-insulation cover thickness L6 (Figure 4) on the temperature on the boundary between the tissue surface and the transducer pad are shown in Figure 8.

The physical experiment with the transducer and phantom was repeated 10 times. The measured sensors’ average temperatures were *T*_1_ = 30.367 ± 0.0091 °C, *T*_2_ = 30.161 ± 0.0096 °C, *T*_3_ = 30.199 ± 0.0087 °C, and *T*_4_ = 29.4521 ± 0.0108 °C. Temperature *T_cp_* on the boundary between the tissue surface and the transducer pad was calculated using Equation (2) and the values of the measured temperatures for *T*_1_, *T*_2_, *T*_3_, and *T*_4_. The estimated temperature *T*_sp_ of the experiment was 30.199 ± 0.0494 °C. The average temperature measured with the two thermistors located on the transducer can be calculated as *T*_a_ = (*T*_1_ + *T*_3_)/2, and it was *T*_a_ = 30.282 °C. The difference is *T_a_* − *T_sp_* = 0.082 °C. The predicted temperature at the depth of 3 cm in phantom was calculated by using Equation (1) and estimated temperature value *T*_cp_ was 33.58 °C.

The average deep-muscle temperature at a depth of ~3 cm before cooling measured by needle thermometer was 36.62 ± 0.147 °C and after cooling was 32.48 ± 0.117 °C; estimated by transducer before cooling was 36.489 ± 0.262 °C and after cooling was 32.32 ± 0.098 °C. The difference of average temperature values estimated by needle thermometer and transducer before cooling was ±0.191 °C and after cooling was ±0.168 °C. Correlation coefficient between temperature values measured with needle thermometer and using transducer before cooling was *r* = 0.723 and after cooling was *r* = 0.945.

## 4. Discussion

In the present study, we showed that temperature in the deeper tissue layers during cooling can be measured using transducer with two thermal channels and four precise thermistors. Results of simulation and physical experiment revealed the importance of thermal properties of thermal channels and thermo-isolation cover materials. The present research used the same steps to develop gear for the temperature measurement at a muscle depth of 3 cm, and this is typical for researchers investigating changes in muscle endurance and responses and workload during muscle cooling, as well as controlling the effects of the treatment by cooling ice packets or other freezing agents.

In our opinion, it is complicated to use diffuse optical spectroscopic imaging method [45] or microwave scanning [39] equipment for temperature measurement during cooling in cold water bath or providing therapy with ice packs after trauma. In study [33], it was shown that when the skin temperature was measured with thermocouple without thermo-isolation enclosure, its value was a weak predictor of intramuscular temperature during cooling.

The transducer developed in our study actually measured the skin temperature (on border skin surface and transducer pad) and after calculation could predict the temperature in deeper layers of muscle. So, could other skin surface sensors be used to measure (predict) temperature in the deeper layers of the tissue during cooling?

For skin temperature measurement, various studies used thermocouples [6,33,37], infrared sensors [27,32], thermistors [29], with the number of types being 147, as specified in a review article [30]. Other studies [29,55,56,57] showed that the measurement of the skin surface temperature with contact sensor was influenced by the ambient temperature, the sensor mounting method, and the method and quality of the thermal insulation of the sensor from the environment.

In our studio, thermo-isolation cover parameters and selected materials with the most suitable thermal conductivity had been optimized in order to protect the developed transducer better against environmental influences. In addition, from the available literature, we did not find any information on the measurements of temperature in deeper tissue layers using skin sensor by cooling the tissue (human limbs) for comparison.

During this study, transducer thermistors’ impedance was measured with a high-accuracy gauge Fluke-type Black Stack used in laboratory environments. For practical measurements in the transducer design, less precise, inexpensive ADC converters and a microprocessor can be used. However, it is likely to achieve less accuracy.

In a previous study [55], the temperature probe of the thermometer (accuracy ± 1 °C) was inserted through the subcutaneous fat layer 1 cm into the muscle, and after cooling for 5 min with an ice pack, the temperature was 34.9 ± 1.2 °C. The inter-muscle temperature at a depth of ~3 cm was measured in Reference [58], but they cooled the whole body at 14 ± 1 °C and the temperature decreased during cooling by 6.96 ± 2.3 °C after 170-min cooling time. Authors of Reference [34] inserted thermocouple at a depth of the skinfold thickness +2 cm and used ice bags for cooling. In Reference [4], inter-muscle temperature was measured at a depth of 1 cm below the skin, and after 20 min of treatment with ice, the temperature decreased to 7 °C. In the study [32], subjects were exposed to −110 °C chamber for 4 min and 8 °C cold water immersion therapy. Correspondingly, the inter-muscle temperature decreased at 3 cm depth by 1.6 ± 0.6 °C and 1.7 ± 0.5 °C. Various ice bag fixing methods were used during the cold treatment [6], and inter-muscle temperature was measured at a depth of 2 cm by thermocouple. The temperature drop in the 30-min time was, from initial value, from 5.6 °C to 10.06 °C depending on the method of the ice bag fixing.

Based on the investigation results mentioned above, it can be stated that the temperature of the deeper muscle layers was measured, in most cases, in an invasive manner, but the results are difficult to compare with each other, because different types of cooling techniques and cooling temperatures were used, and the temperature was measured at various tissue depths.

In the future studies, the gear for temperature in deeper layers of the muscle during cooling measurement should be designed with transducer, software, microcomputer, and display. Studies should be conducted with a larger number of subjects and in both sexes (different skinfolds), and the measurement accuracy should be estimated.

## 5. Conclusions

Using simulation results, the experimental transducer was designed for the temperature measurement in deeper tissue layers during cooling. The temperature measurement with the needle thermometer and the transducer results were well correlated as before tissue cooling *r* = 0.723 and after cooling *r* = 0.945, and the temperature difference was no more than ±0.2 °C.

Currently, there are no commercially available sensors for measuring temperatures in deeper muscle layers during cooling human’s limbs or for temperature control at a certain depth of tissue during treatment time with ice bags after trauma.

After commercialization, the developed transducer can be recommended for use in studies of muscles properties change during cooling and for temperature control in the treatment with ice bags after trauma.

## Figures and Tables

**Table 1:** Human tissue layer properties.

Layer	*ρ* (kg/m^3^)	*k* (W/m*K)	*Cp* (J/kg*K)	Thickness (m)	*Q_m_* (W/m^3^)	*ω_b_* (1/s)	*T_b_* (K)
Muscle	1090	0.5	3766	0.12	5	0.0001	310.15
Fat	850	0.16	2510	0.0025	0	4/5E−6	310.15
Skin	1100	0.21	3250	0.002	4	7.2E−8	310.15
Blood	1050	0.32	1313	0.03		0.5	310.15
